# Implantable Impedance Plethysmography

**DOI:** 10.3390/s140814858

**Published:** 2014-08-13

**Authors:** Michael Theodor, Dominic Ruh, Martin Ocker, Dominik Spether, Katharina Förster, Claudia Heilmann, Friedhelm Beyersdorf, Yiannos Manoli, Hans Zappe, Andreas Seifert

**Affiliations:** 1 Department of Microsystems Engineering, University of Freiburg, Georges-Koehler-Allee 102, 79110 Freiburg, Germany; E-Mails: michael.theodor@imtek.de (M.T.); dominic.ruh@imtek.de (D.R.); martinocker@arcor.de (M.O.); dominik.spether@imtek.de (D.S.); ymanoli@imtek.uni-freiburg.de (Y.M.); Hans.Zappe@imtek.uni-freiburg.de (H.Z.); 2 Heart Center Freiburg University, Hugstetter Strasse 55, 79106 Freiburg, Germany; E-Mails: katharina.foerster@uniklinik-freiburg.de (K.F.); claudia.heilmann@uniklinik-freiburg.de (C.H.); friedhelm.beyersdorf@uniklinik-freiburg.de (F.B.); 3 HSG-IMIT, Institute of Micromachining and Information Technology, Wilhelm-Schickard-Strasse 10, 78052 Villingen-Schwenningen, Germany

**Keywords:** biomedical implant, arterial distension, plethysmography, blood pressure

## Abstract

We demonstrate by theory, as well as by *ex vivo* and *in vivo* measurements that impedance plethysmography, applied extravascularly directly on large arteries, is a viable method for monitoring various cardiovascular parameters, such as blood pressure, with high accuracy. The sensor is designed as an implant to monitor cardiac events and arteriosclerotic progression over the long term.

## 1. Introduction

Over one billion people worldwide suffer from hypertension, which is the leading global risk for mortality [1]. Reliable blood pressure (BP) management is the key to avoiding cardiac events resulting from hypertension. The regular measurement of blood pressure, outside of the clinical environment, can optimize therapy in terms of drug delivery [2]. In particular, the pressure profile over an entire night should be recorded to characterize dangerous nocturnal hypertension [3].

Continuous telemetric monitoring of cardiovascular parameters would deliver the fast identification of emergencies and, hence, would enable optimized therapy. Individualized optimization of medication based on the analysis of long-term data from home monitoring will definitely result in an increased life quality. By long-term monitoring, cardiovascular events, such as heart attack or stroke, can be detected at a very early stage, and the consequential damages can be reduced by fast emergency interventions. Clinical studies [4,5] confirm that telecardiology reduces mortality in comparison with standard care. Moreover, it has been shown that telecardiology can decrease the number of hospitalizations, as well as the time a patient has to stay in the hospital [4]. In total, it is expected that the costs for healthcare are reduced [2], particularly with respect to the demographic change leading to an older population [6].

The problem with state-of-the-art techniques for monitoring blood pressure is the lack of devices that may be used by a mobile patient undertaking the activities of everyday life. The standard methods currently used to measure blood pressure are predominantly intra-arterial catheters and cuff-based oscillometric monitors. For the catheter, the drawbacks are its invasiveness, leading to risks outside of the clinical environment and for the cuff-based system, inconvenience and inaccuracies. As a result, both methods have proven to be impractical for long-term monitoring without limiting the patients' mobility.

To overcome these drawbacks, a minimally-invasive implantable sensor may be used. As the demand for such a sensor is high, the first approaches have been published to measure blood pressure extravascularly directly at an artery by mechanical [7,8], optical [9] and magnetic [10] methods.

This paper presents a new method to detect the arterial pulse wave by an extravascularly implantable approach using impedance plethysmography. It is based on measurements of the vascular impedance yielding the diameter of the blood vessel. The sensor has shown promising results during *ex vivo* and *in vivo* trials. Theory, simulations and measurements underscore the method's capability of detecting arterial expansion and its strong correlation to blood pressure.

## 2. Theory

The electrical resistance of the human body has been widely studied. Impedance cardiology is the non-invasive visualization of the motion of the heart and has been performed since the 1960s. It is a tomographic method measuring the body's impedance, using several electrodes around the chest. The concept relies on the good conductivity of blood compared to tissue and bones [11].

Impedance plethysmography (IPG) is used extra-corporeally to measure perfusion, the arterial pulse wave, as well as venous occlusions. For the measurement, four ECG electrodes are applied to the patient's arm or leg to measure its impedance. Similar to pulse oximetry, the surrounding tissue generates an unwanted offset in the sensor signal, decreasing the signal-to-noise ratio of the method. As extracorporeal IPG cannot distinguish between arterial and venous blood, it is not the recommended method for pulse wave analysis and pulse transit time determination.

**Figure 1.**
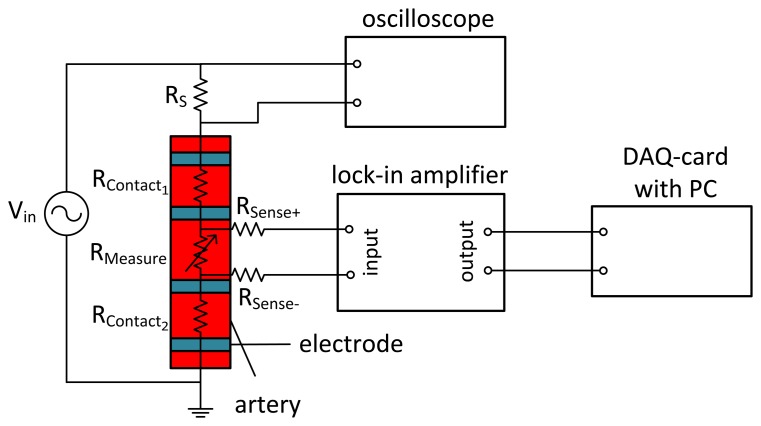
Four-wire measurement of arterial impedance. An alternating voltage (*f* = 100 kHz) is applied to the outer source electrodes. The voltage at the sense electrodes, demodulated by the lock-in amplifier, is only influenced by the impedance of the artery.

The idea of applying an IPG sensor directly on an artery is pursued in our research presented in this paper. Around 1989, Apfel *et al.* performed basic impedance measurements directly on an artery [12–16]. The direct application of the electrodes to a blood vessel separates its resistance from that of tissue and veins. To exclude the contact resistance, a four-wire measurement, as shown in Figure 1, is realized, where the two outer electrodes are the source contacts and the two inner electrodes represent the sense contacts. As the contact resistance between electrodes and arteries is lower for AC signals, alternating currents are used. The frequency *f* has to be chosen with care, since the conductivity of skin drastically decreases with frequencies in the MHz and GHz range. However, frequencies below 1 kHz interfere with the nervous system, trigger muscle contractions and cause cardiac dysrhythmia [17,18]. As a compromise, commercially available devices use frequencies between 20 and 100 kHz [11,19].

In a simple model of a cylindrically-shaped electrical conductor with blood as the main conductor and neglecting the influence of surrounding tissue, the arterial impedance is given by:

(1) $$Z_{\text{artery}}(t)=\frac{V(t)}{I_{0}}=\rho_{\text{blood}}\cdot \frac{l}{A(t)}=\rho_{\text{blood}}\cdot \frac{l}{\pi {\left(d(t)/2\right)}^{2}}$$

Here, *A*(*t*) is the inner arterial cross-sectional area, *ρ_blood_* the resistivity of blood, *I*_0_ the applied alternating current with constant amplitude, *l* the length between the sense electrodes and *d*(*t*) = 2*r*_1_(*t*) the varying inner blood vessel diameter. The resistivity for the materials applied is given in Table 1. Since all variables of Equation (1) are either known constants or given by design, the measured voltage *V* (*t*) directly leads to the inner blood vessel diameter:

(2) $$d(t)=\sqrt{\frac{4\cdot \rho_{\text{blood}}\cdot l\cdot I_{0}}{\pi }}\cdot \sqrt{\frac{1}{V(t)}}$$

The model can easily be extended by a second cylindrical tube, such as the arterial wall, thereby approximating the *ex vivo* configuration. The corresponding total impedance results then from the parallel circuit of the inner cylinder (blood) and the outer tube (vessel wall) as:

(3) $$Z_{\text{tot}}(t)=\frac{l}{\pi }{\left(\frac{r_{1}{\left(t\right)}^{2}}{\rho_{\text{blood}}}+\frac{r_{2}{\left(t\right)}^{2}-r_{1}{\left(t\right)}^{2}}{\rho_{\text{wall}}}\right)}^{-1}$$

with *ρ_wall_* as the resistivity of the arterial wall and *r*_2_ as the outer radius of the artery, including the wall. As in Equation (2) for the simple model, we can directly derive the inner radius of the artery from the extended model of Equation (3),

(4) $$r_{1}=-t_{\text{wall}}\frac{\rho_{\text{blood}}}{\rho_{\text{wall}}}+\sqrt{t_{\text{wall}}^{2}\frac{\rho_{\text{blood}}^{2}}{\rho_{\text{wall}}^{2}}-t_{\text{wall}}^{2}\frac{\rho_{\text{blood}}}{\rho_{\text{wall}}}+\frac{l\rho_{\text{blood}}}{\pi Z}}$$

where *t_wall_* = *r*_2_ − *r*_1_ is the thickness of the arterial wall. From Equation (4), we see that the inner radius of the artery can be determined from measured impedances if the wall thickness is known.

In reality, the exact value of *t_wall_* is often not known, but it can be estimated with an uncertainty Δ*t_wall_*. Assuming an uncertainty of Δ*t_wall_* = *±*100 μm, the error of the absolute value of the inner arterial radius is about *±*31 μm. Here, typical physiological values of *r*_1_ = 2 mm and a mean wall thickness of *t_wall_* = 700 μm have been taken, and the distance between the sense electrodes is *l* = 2 cm. The uncertainty is calculated by Gaussian error propagation applied to Equation (4) and refers to the absolute values of the radius. However, if we look to the relative changes of the inner radius Δ*r*_1_, as they occur during a cardiac cycle or due to mean pressure variations, the uncertainty of *t_wall_* will cause only a small error in Δ*r*_1_ in the order of 4 μm for arterial distensions of 10 %.

## 3. System Design

### 3.1. System for Acute Measurements

**Figure 2.**
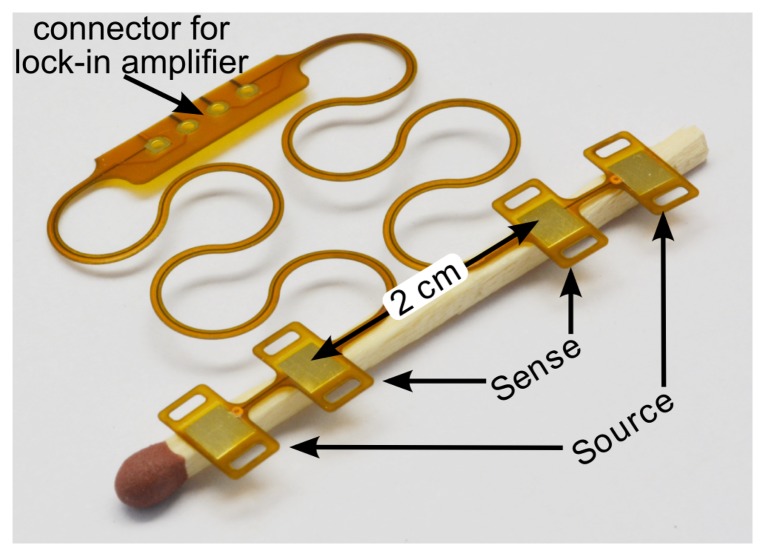
Flexible polyimide foil with four electrodes to be placed on an artery. The meander structure makes the connection stretchable.

A system consisting of four flat 10-mm^2^ copper electrodes has been designed, as shown in Figure 2. The substrate is a flexible PI (polyimide) foil featuring horseshoe-shaped structures to allow stretchability, as proposed by [21]. This system is used for cable-based measurements.

The electrodes used for the impedance measurements are designed to be on the same side of the artery, with distances 3.5 cm between the source electrodes and 2 cm between the sense electrodes. In an optical approach to measure arterial distention, proposed by Ruh *et al.* [22], the sensor and detector are placed diametrically on opposite sides of the artery. For the IPG measurement, a diametric configuration would lead to a inhomogeneous current distribution in the artery with worse sensitivity than the axial electrode separation used here.

An AC voltage source is applied to the source contacts, causing an AC current to flow through the arteries. The heartbeat, and hence, the pressure wave in the artery, causes a radial distension of the blood vessel wall, which modulates the amplitude of the alternating signal. With the sense contacts, the potential difference is measured and amplified by a lock-in amplifier.

### 3.2. Implantable System

The main limitations on an implant are its size, power consumption and the amount of transmitted data. State-of-the-art lock-in amplifiers consume too much current and need too much space to be an implantable solution.

**Figure 3.**
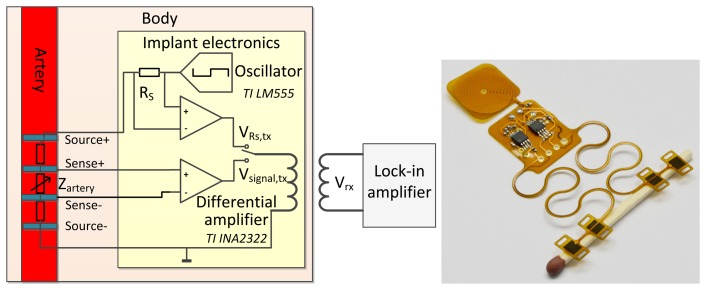
(**Left**) Schematic of a battery-powered implantable version of the impedance plethysmography (IPG) sensor. The modulated voltage at the sense contacts is conditioned by a differential amplifier and inductively coupled to the extracorporeal lock-in amplifier. (**Right**) Photograph of this circuit, realized on flexible polyimide foil.

To overcome these problems, we propose to wirelessly transmit modulated analog data. The implantable system is battery-powered and generates the AC signal for the measurement, as presented in the schematic in Figure 3. The signal from the sense electrodes is conditioned by a differential amplifier and applied to a transmitting coil. The system is inductively coupled to an extracorporeal receiver coil, connected to the lock-in amplifier. The signal strength is highly influenced by the inductive coupling coefficient *k*, which depends on the distance and orientation of the coupled coils. To avoid the drawback of the influence of the inductive coupling coefficient on the measurement result, the voltage across the 10 kOhm shunt resistor *R_S_* can also be transmitted. The actual impedance of the arterial segment *Z_artery_* can be calculated from the measured signal *V_signal,rx_* and the voltage drop over *R_S_* , *V_R_S_,rx_*, which are both linearly influenced by *k*. Due to the linear influence of *k*, the ratio of *V_signal,rx_*(*k*) and *V_R_S_,rx_*(*k*) is not dependent on the distance between the coupled coils. The arterial impedance can be calculated by:

(5) $$Z_{\text{artery}}=R_{S}\cdot \frac{V_{\text{signal},\text{rx}}(k)}{V_{R_{S},rx}(k)}$$

leading to the inner diameter of the blood vessel by either Equation (2) or Equation (4).

## 4. Simulation

**Figure 4.**
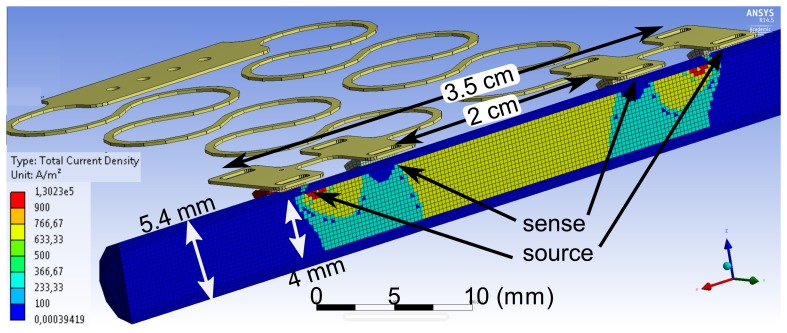
FEM simulation of the current density inside an artery of a 4-mm diameter with a vessel wall thickness of 700 μm. A current of 10 mA is fed to the outer source electrodes.

The sensing principle has been simulated using ANSYSFEM tools. An alternating current is injected at the outer electrodes, and the voltage is measured between the inner sense electrodes. Figure 4 shows the simulated current density inside an an artery using the material parameters from Table 1. The inner vessel diameter is set to 4 mm, with a realistic wall thickness of 700 μm. In the simulation, saline is taken as the liquid in the tube, providing the same conditions as in the *ex vivo* experiment. It can be seen that the current is distributed uniformly inside the artery between the inner sense electrodes. The current density inside the arterial wall is a factor of six smaller. The simple model of Equation (1), neglecting the arterial wall, yields an impedance of 1.003 kΩ between the sense electrodes separated by 2 cm. The simulation result under consideration of the entire geometry, including the vessel wall, gives 963 Ω. The analytical description of the extended model of Equation (3) gives 923 Ω, hence underestimating the actual value. The deviation between the simulation, which is more realistic, and the analytical Equations (1) and (3) can be explained by the different ways of injecting the current. The analytical descriptions assume a full cross-sectional contact, whereas in reality, and in the simulation, only small contacts are connected to the cylinder surface.

**Figure 5.**
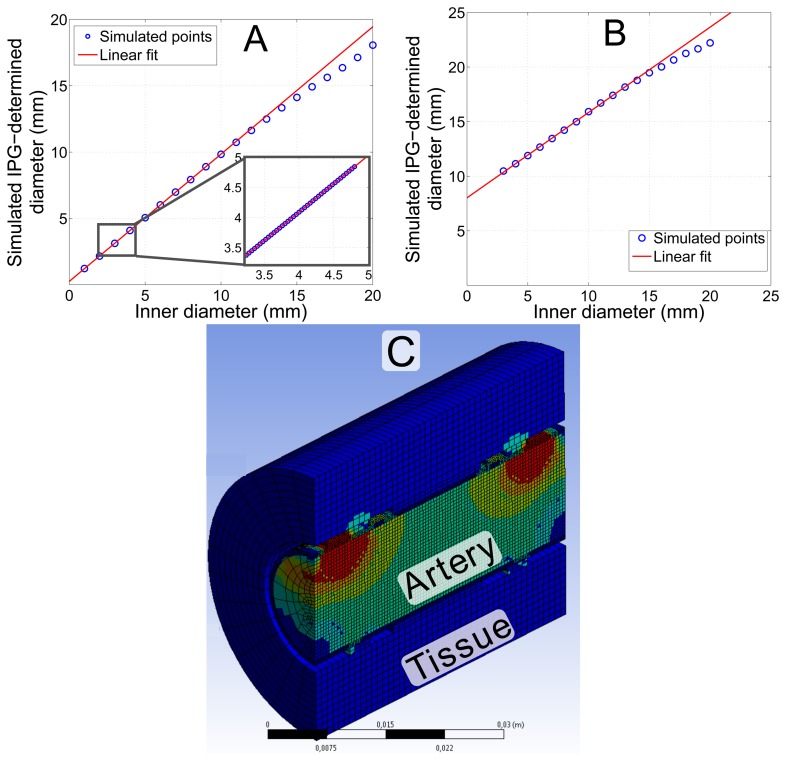
Simulation of the diameter, determined by IPG measurements using the FEM model shown in Figure 4. (**A**) Simulation of an extended range of the arterial diameter for an *ex vivo* artery. The inset magnifies the physiologically most relevant part with a correlation of *r* = 1.0. The slope of the linear fit is *m* = 0.96 and the offset *c* = 0.25 mm. (**B**) Simulation considering *in vivo* conditions with 20 mm of tissue around the artery. The slope of the linear fit is *m* = 0.78 and the offset *c* = 8 mm; correlation coefficient *r* = 0.9997. (**C**) The model considering *in vivo* conditions with additional layers of tissue.

To prove the validity of Equation (2), the arterial diameter *d*(*t*) was varied and the voltage at the sense electrodes simulated. Vessel wall compression was modeled with a Poisson's ratio of *ν* = 0.5. Figure 5A shows that Equation (2) holds with almost an ideal correlation of *r* = 0.99996 for a diameter between 1 and 10 mm. This result proves that even the simplified model of Equation (2) appropriately describes the measurement principle for an *ex vivo* configuration. According to theory, the values represent the inner instead of the outer diameter. Larger diameters lead to an inhomogeneous current density distribution between the electrodes and, hence, larger deviation from theory. The method is intended to be applied to the carotid, femoral, radial, brachial or iliac artery, which all have diameters below 10 mm. Accordingly, deviations from the theoretical description are not expected.

To simulate *in vivo* conditions, the influence of tissue, with data from Table 1, surrounding a real artery was considered. The copper electrodes are not in direct contact, but capacitively coupled to the tissue. The artery is wrapped in a 2-mm layer of fat, mimicking connective tissue. An outer layer of 20 mm of muscle tissue was included on top of the connective tissue. Figure 5B shows that the tissue leads to a clear overestimation of the diameter. However, the most important support for the measurement method is the fact that the system still shows linear behavior with *r* = 0.9997. Even large (5 mm) simulated changes of the surrounding tissue lead to only a small offset in the sensor signal of 106 μm. Tissue that is more than 2.5 cm away from the artery does not influence the signal in this simulation geometry.

To compensate for the influence of the surrounding tissue, a calibration has to be performed. The arterial diameter can be determined non-invasively from existing ultra-sound measurement methods with sufficient accuracy [23]. However, these non-invasive ultra-sound devices are not suitable for the continuous monitoring of a mobile patient.

## 5. Measurement Results

### 5.1. Ex Vivo Measurement

#### 5.1.1. Measurement Setup

**Figure 6.**
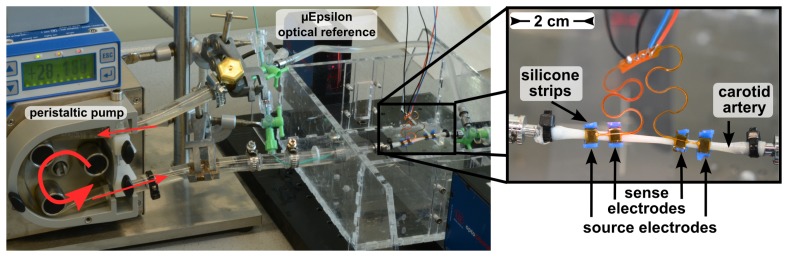
Setup of the *ex vivo* measurement with a carotid artery of a domestic pig in an artificial circulatory system. The IPG electrodes are mounted on the artery using flexible silicone strips.

For the *ex vivo* evaluation of impedance plethysmography on an artery, the setup shown in Figure 6 was employed. The electrodes with areas of 10 mm^2^ are mounted on a pig's carotid artery with an outer diameter of 4.5 mm and a length of 6 cm, using PMDS strips around the artery for mounting. The spacing between the sense electrodes was 2 cm and between the source electrodes 4 cm.

A peristaltic pump was used to generate pulses at a frequency of 1.2 Hz. Flexible tubes connect the pump with the artery. Normal saline with 0.9% NaCl was used as the liquid in the circuit. The signal at the sense electrodes was demodulated by a SRS SR830 DSP lock-in amplifier, low-pass filtered with a cut-off frequency of 100 Hz and recorded with a DAQcard at 1 kHz. The absolute outer diameter was detected with a precision of 1 μm by an optical measurement method (Micro-Epsilon optoCONTROL 2600), which measures the size of the shadow of the artery, as a reference.

#### 5.1.2. Optimization of Control Parameters

To determine suitable parameters for the final measurements, we first vary and optimize the modulation frequency and, second, the source current.

The influence of the modulation frequency on the mean impedance is shown in Table 2. Each measurement point has a standard deviation of ±5 Ω. The total variation between 1 and 100 kHz is 4%. The signal-to-noise ratio and the relation to arterial distension also remain almost constant for varied frequencies. For the following measurements, 100 kHz is used as the modulation frequency.

**Figure 7.**
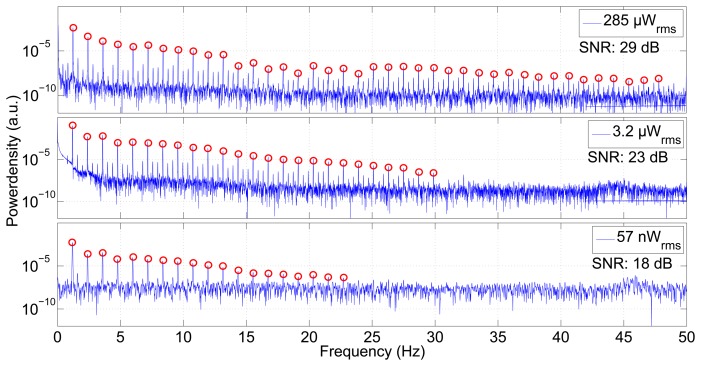
Power spectral density of the measurement shown in Figure 8. The three plots differ in the current fed into the source electrodes. Higher current and, hence, higher power produces an excellent signal-to-noise ratio, making the pumping frequency and more than 40 harmonics visible. Even a very low power of 57 nW reveals the pumping rate and 18 overtones. The signal-to-noise ratio (SNR) decreases with lower power.

The impedance was measured using different supply currents. Figure 7 shows the power spectral density of the measured impedance signals. These spectra show the pumping frequency of 1.2 Hz and its overtones. The better the signal-to-noise ratio of the method, the more overtones are visible. Using an average power of 285 μW for the sensor yields a signal-to-noise ratio of 29 dB and makes more than 40 harmonics visible, whereas a power of 3.2 μW gives 24 overtones with an SNR of 23 dB. Even with the extremely low source power of 57 nW, a clear signal, still with a good SNR of 17 dB and 18 visible overtones, can be measured.

#### 5.1.3. Results

The pulses measured by arterial impedance are compared with the pulsatile variation of the arterial diameter for constant modulation frequency and source current.

**Figure 8.**
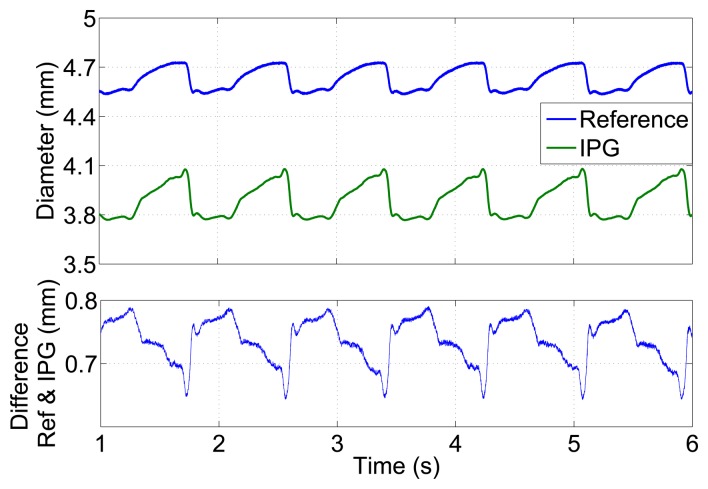
Diameter *versus* time for a carotid artery of a domestic pig in an *ex vivo* measurement in an artificial circulatory system measured by the optical *μ*Epsilon reference and by impedance plethysmography. The lower plot shows the deviation between both plots.

The arterial diameter, calculated from the unfiltered IPG signal (*f_m_* = 100 kHz, *P_source_* = 285 μW), is shown in Figure 8 and demonstrates IPG-pulses with a very low noise level. Compared to the outer vessel diameter measured by the reference system, the values are 600 to 800 μm smaller. As the simple model of Equation (2) for calculating the diameter only considers the well-conducting saline inside the vessel and does not take into account the vessel wall, it only yields an approximation of the inner vessel diameter. The lower plot in Figure 8 shows the varying deviation caused by the vessel wall thickness over time. Larger expansions show a smaller deviation, due to the compression of the vessel wall.

**Figure 9.**
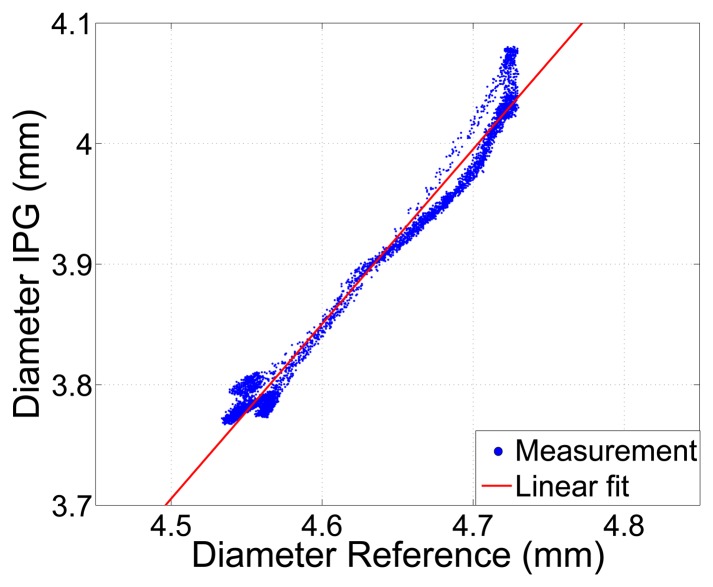
Arterial diameter determined by IPG, using Equation (2) *vs.* a reference measurement of the outer diameter. Correlation coefficient *r* = 0.99.

Despite the offset, the vessel diameters determined by the IPG measurement by applying Equation (2) and the reference measurement show a linear relationship, as shown in Figure 9, with an excellent correlation coefficient of *r* = 0.99. This verifies, on the one hand, the simulation and, on the other, the simplified theory and makes the system capable of tracking variations of inner arterial diameters.

### 5.2. In Vivo Measurement

*Ex vivo* measurements on isolated arteries do not represent the realistic *in vivo* scenario. The question now is how the system behaves *in vivo* with different surrounding layers of tissue. As we saw above, simulation predicts that the presence of tissue will influence the slope, but not the linearity of the characteristic. The measurement principle was tested *in vivo* in a domestic pig (male, six months old, body weight 103 kg) during surgery. Anesthesia was maintained with propofol, vecuronium and fentanyl, while the volume controlled ventilation was set to 14 breaths/min (650 mL tidal volume). Blood pressure was monitored by a tip catheter injected in the femoral artery. A reference to measure arterial diameter was not available.

**Figure 10.**
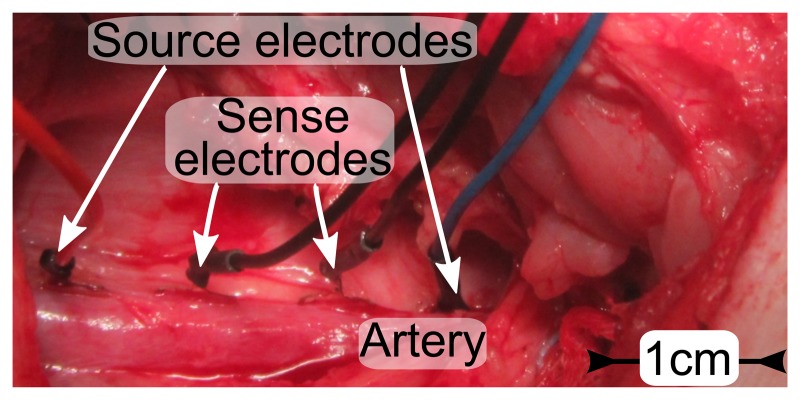
Photo of electrodes placed on the femoral artery (Ø 9 mm) of a domestic pig. The spacing is 1 cm between sense electrodes and 3 cm between source electrodes.

The four electrodes with a size of 10 mm^2^ were positioned on the femoral artery, which had a diameter of 9 mm. As shown in the photograph in Figure 10, all electrodes are placed on the same side, between artery and tissue, attached only by contact pressure, without silicone strips or suture. The spacing between the sense electrodes is 1 cm and 3 cm between the source electrodes. As in Figure 1, the lock-in amplifier is connected by cables. The modulation frequency is 100 kHz, and an analog low-pass filtering is set at 1 kHz. Measurement data are acquired by a National Instruments USB-6251 DAQ-card with a sampling rate of 10 kHz.

**Figure 11.**
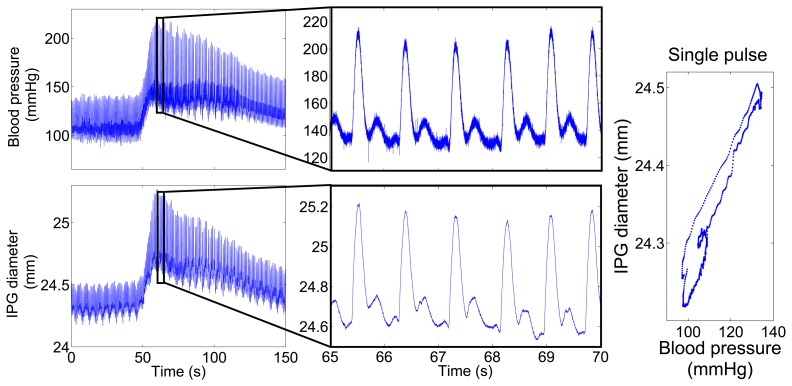
Measured blood pressure and IPG-determined diameter over time. After 50 s, the blood pressure was increased by a bolus of 50 μg noradrenaline. (**Left**) The complete measurement over 150 s. (**Middle**) Plot zoom into 5 s of the same measurement to visualize the blood pulses with a heart rate of 70/min. (**Right**) The plot displays blood pressure *vs.* IPG-signal for a single pulse.

Figure 11 shows the IPG-determined diameter, calculated by Equation (2), and the intra-arterial pressure of the same artery for 150 s. Blood pressure was increased by a bolus injection of 50 μg noradrenaline after 50 s. In agreement with the simulation shown in Figure 5B, the absolute values of the diameter are overestimated, due to surrounding tissue and the liquid inside the wound. However, the data reveal a clear correlation of blood pressure and IPG data. As expected, the noradrenaline bolus leads to a rise in blood pressure, as well as in diameter. The magnified signal on the right of Figure 11 displays a very similar curve shape for the pulses measured by IPG and blood pressure. The IPG-signals are not digitally filtered, but are still almost noise-free.

**Figure 12.**
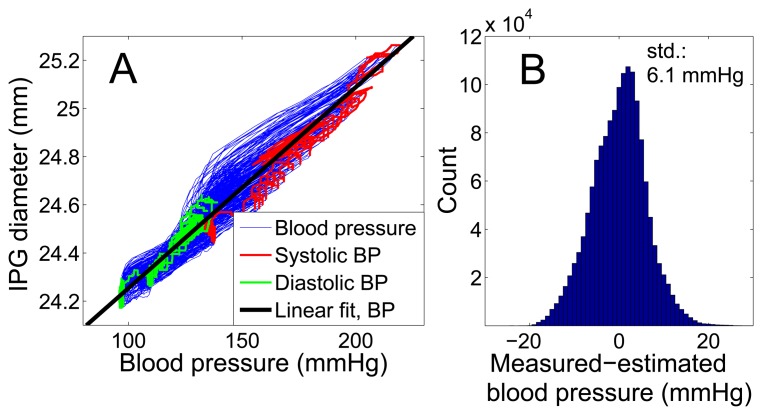
Comparison of intra-arterial blood pressure and vessel expansion measured by IPG for 200 blood pulses. (**A**) Direct comparison of the entire 150 s of the measurement data shown in Figure 11 with a linear fit curve, which is used for calibration. (**B**) The histogram shows the deviation of IPG-determined blood pressure for the same measurement, using the calibration curve from (A). The standard deviation is 6.1 mmHg.

Figure 12 demonstrates the linear relation between the IPG-determined diameter and blood pressure with a high correlation coefficient of *r* = 0.96 for the large pressure variation of 120 mmHg. The determination of blood pressure by IPG requires a calibration, which could be done both either intracorporeal or extracorporeal. For the extracorporeal calibration of an implant, we recommend to at least monitor the patient over a period of 24 h to cover a large blood pressure range. The error of the calibration method and the blood pressure range will influence the accuracy of the system, as described in [24].

Using the intracorporeally determined linear fit of Figure 12 for the calibration, a standard deviation (std) of 6.1 mmHg (4.7 %) and a mean deviation of 4.8 mmHg (3.7 %) for IPG-determined blood pressure results. These values comprise all points on the blood pressure curve, not only systolic or diastolic pressure. Table 3 shows that for this measurement, the sensor can be classified as a Grade A blood pressure monitor, which is the top grade according to the standards of the British Hypertension Society [25]. Concentrating solely on the systolic blood pressure values, a higher precision of std = 4.4 mmHg (2.6 %) results.

## 6. Conclusions

Impedance plethysmography on large arteries as a new method for diagnosing cardiovascular parameters was introduced by theory and simulation, and its validity has been demonstrated *ex vivo*, as well as *in vivo* in a domestic pig. The impedance measurements show a high correlation with arterial blood pressure, a sensitivity that allows reliable measurements with good resolution and an excellent signal-to-noise ratio. The method promises to quantify the dynamics of the inner arterial diameter as an important measure for arteriosclerosis. After calibrating the system, the sensor performance achieves the highest level for measuring blood pressure defined by the British Hypertension Society. Due to the low power consumption, the system shows the potential to be used as an implant for the long-term monitoring of cardiovascular parameters in daily routines for high risk patients.

## Figures and Tables

**Table 1.** Resistivity of biological materials [20].

**Material**	**Blood (37 °C)**	**Saline (22 °C)**	**Arterial Wall**	**Fat**
resistivity *ρ*	1.5 Ω*·*m	0.63 Ω*·*m	6 Ω*·*m	20 Ω*·*m

**Table 2.** Arterial impedance at different modulation frequencies. The measured arterial segment is 20 mm long and 4.5 mm in diameter.

**Modulation Frequency (kHz)**	1	10	50	100
**Mean Impedance (Ω)**	1,140	1,132	1,156	1,175
**Signal-to-Noise Ratio (dB)**	28	28	28	26

**Table 3.** Sensor performance according to the grading of the British Hypertension Society. Blood pressure sensors in Categories A and B are recommended.

	**< 5 mmHg**	**< 10 mmHg**	**< 15 mmHg**
IPG-determined blood pressure	A: 61%	A: 89%	A: 98%
